# Evaluation of bacterial leakage and marginal adaptation of the bioceramics pulp dressing materials: an invitro study

**DOI:** 10.1186/s12903-023-03129-1

**Published:** 2023-07-08

**Authors:** Niher Tabassum Siddiqua Snigdha, Aimi Kamarudin, Fadzlinda Baharin, Nik Rozainah Nik Abdul Ghani, Mohd Firdaus bin Yhaya, Wan Muhamad Amir W Ahmad, Mohmed Isaqali Karobari

**Affiliations:** 1 Department of Paediatric Dentistry, School of Dental Sciences, Universiti Sains Malaysia, Health Campus, Kubang Kerian, Kelantan 16150 Malaysia; 2 Department of Conservative Dentistry, School of Dental Sciences, Universiti Sains Malaysia, Health Campus, Kubang Kerian, Kelantan 16150 Malaysia; 3 Department of Biomaterials and 3D Imaging (BioM3D) Laboratory, Universiti Sains Malaysia, Health Campus, Kubang Kerian, Kelantan 16150 Malaysia; 4 School of Dental Sciences, Universiti Sains Malaysia, Health Campus, Kubang Kerian, Kelantan 16150 Malaysia; 5 Department of Restorative Dentistry & Endodontics, Faculty of Dentistry, University of Puthisastra, Phnom Penh, 12211 Cambodia; 6 Department of Conservative Dentistry & Endodontics, Saveetha Dental College and Hospitals, Saveetha Institute of Medical and Technical Sciences, Chennai, Tamil Nadu 600077 India

**Keywords:** Bioceramics dressing material, Biodentine, Mineral trioxide aggregate, ProRoot, Coronal pulpotomy, Bacterial leakage test, Scanning electron microscope

## Abstract

**Objective:**

To compare the sealing ability and marginal adaptation of three calcium silicate-based cement (Biodentine, Pro root MTA, MTA Angelus) using a bacterial leakage model and scanning electron microscope (SEM).

**Methods:**

Recently extracted lower first premolars were randomly categorized into three experimental groups (n = 15 samples), positive control (n = 5 samples), and negative control group (n = 5 sample). Samples from the experimental groups and positive control group were subject to cavity Class I occlusal preparation followed by modified coronal pulpotomy. Different types of bioceramic dressing material were placed in 3 mm thickness accordingly, group 1 (Biodentine), group 2 (MTA Angelus), and group 3 (ProRoot MTA). No dressing material was placed in the positive control group (group 4). All samples were placed in the incubator for 24 h at 37℃, 100% humidity, for the materials to be completely set. The final restoration was placed using the Z350 resin composite. A double layer of nail varnish was applied over all the sample surfaces except the occlusal site. Whereas the samples’ surfaces in the negative control, were completely covered. A 3 mm length was measured from the root apex of the samples from each group, before proceeding with the resection. The bacterial leakage test was performed using *Enterococcus faecalis TCC* 23,125, and a sample from each experimental group was randomly chosen for SEM. Data analysis was conducted under the One-way ANOVA test, completed by Tukey’s post hoc test.

**Results:**

There is a significant difference in sealing ability and marginal adaptation between the groups. (p < 0.05). The study showed that Pro Root MTA had the superior sealing ability and marginal adaptation compared to Biodentine and MTA Angelus.

**Conclusion:**

The ProRoot MTA as a coronal pulpotomy pulp dressing material, was found to have a better marginal adaptation and sealing ability compared to three other bioceramics materials. The material would be the better choice during clinical settings and procedures.

## Introduction

Pulpotomy is a clinical treatment where the coronal segment of the vital pulp tissue is surgically amputated, whereas the pulp radicular area is preserved [1]. A biocompatible wound-dressing material is placed on the amputated coronal pulp and seals the remaining radicular pulp tissue against continuous inflammation, infection, or injury. Thus, encourage healing and reparative as part of a regenerative procedure [2]. The pulpotomy procedure can be divided into either partial which is referred to as partial pulpotomy, or a complete pulpotomy which refers to the entire removal of the coronal pulp tissue [3].

In the recent practice of Paediatric Dentistry, complete or coronal pulpotomy procedure can be implemented on immature permanent teeth and primary molars. Coronal pulpotomy is where the wound dressing materials are placed in the healthy radicular pulp tissue before placement of the permanent restoration [2]. Coronal pulpotomy in the immature permanent tooth is capturing more attention from clinicians because of its minimal invasive treatment procedure, which is less complicated and cost-effective compared to other conservative protocols, such as root canal treatment (RCT) [4]. The success ratio of coronal pulpotomy was found to be 82.9 − 100%, and it was highly associated with the type of pulp dressing medicaments used [5]. On the other hand, for over 100 years, it has been known that the main factor in endodontic treatment failure was microbial leakage [6].

The commonly used materials for dressing after amputation of coronal pulp were bioceramics or calcium silicate-based types of cement which include Biodentine and MTA (mineral trioxide aggregate). The use of MTA as pulp dressing material, followed by glass ionomer cement (GIC) and stainless steel crown, gave high clinical and radiographic success. [5] Meanwhile, another study found that the use of Biodentine as a dressing material appears to show a promising result [7]. A study of microbial leakage [8] using multiple bioceramics materials showed that the Biodentine and ProRoot MTA produced the best marginal adaptation and sealing ability. Many clinical studies have been conducted to investigate the effectiveness of different dressing materials in coronal pulpotomy procedures for permanent teeth, but most of them used radiographs and clinical evaluations. Our recent study however was focusing on the MTA Angelus, Biodentine and ProRoot MTA in the scope of sealing ability and marginal adaptation under bacterial leakage test. Scanning Electron Microscope (SEM) was proposed to assess the marginal adaptation and bonding between materials and the wall. Thus, the aim of this study was to compare the sealing ability and marginal adaptation of three calcium silicate-based cements (Biodentine, ProRoot MTA, MTA Angelus) using a bacterial leakage model and SEM.

## Materials and methods

Ethical clearance was obtained as well as approved by the Human Research Ethics Committee (JEPeM) of Universiti Sains Malaysia (USM/JEPeM/22,010,089). The recently extracted mandibular 1st premolars due to periodontal or orthodontic reasons were collected from the Department of Oral and Maxillofacial Surgery, School of Dental Sciences, Universiti Sains Malaysia, Health Campus, Kubang Kerian, Kelantan, Malaysia. The teeth with mature single-root form (the age of patients ranging 20y − 40 y) were included. The size of the sample was calculated using PS Software version no 3(Walton D. Plummer, Jr. and William D. Dupont), with a standard deviation of 2.87 mmol/L, [9], the probability of 0.8 power along with 0.05 alpha value. An additional 15% of samples were included in each group for possible dropout [12] [10]. The total sample desired for this study was 55 (3 experimental groups containing 15 and control groups with 5 samples each) (Table 1).

**Table 1:** The group and allocated materials

Group		Procedure and allocated material
**Experimental**	Group 1	Modified coronal pulpotomy procedure pulp dressed in Biodentine
	Group 2	Modified coronal pulpotomy procedure pulp dressed in MTA
	Group 3	Modified coronal pulpotomy procedure pulp dressed in ProRoot MTA
**Positive control**	Group 4	modified coronal pulpotomy pulp without dressing material.
**Negative control**	Group 5	no intervention

calibrated digital slide caliper was used to ensure the standardized sample length of 21 mm. Furthermore, the ultrasonic scaler (Dentsply Sinora, Germany), was used to remove tissue debris and calculus from the samples. A digital radiograph (Planmeca, Finland) was constructed to confirm the presence of a single canal. All samples were soaked in 2.5% NaOCl (sodium hypochlorite) solution for disinfection for 24 h [10] and later distributed into 3 groups of experimental (n = 15 samples), positive control group (n = 5), and negative control group (n = 5).

### Sample preparation

**Phase 1 – Modified coronal pulpotomy procedure.**

The modified coronal pulpotomy procedure in this study refers to a standardized coronal pulpotomy procedure followed by root canal preparation. The root canal preparation was performed following the coronal pulpotomy procedure to achieve a pure, sterile environment in the teeth sample for the bacterial leakage model. The standardized access cavity of 3 mm x 3 mm was performed for the experimental groups and a positive control group. The high-speed handpiece (Bien Air, Switzerland) with an Endo-Access diamond bur no. 4 (Dentsply, Switzerland) was used for the procedure. The access cavity diameters were measured using a digital caliper. The corrected working length was measured from the tip of the buccal cups to the apical foramen. The root canal preparation was done using the Endo-Eze™ Genius® Motor system (Ultradent, USA) using Sx and S1 (Dentsply Mainllefer, Switzerland) Protaper Rotary files. During preparation, 2.5% Sodium hypochlorite (NaOCl) (Lenntech, Netherlands) was used as an irrigant. In addition, 17% EDTA (Promega Corporation, USA) was used to expel the smear layer, and other chemical remnants [11]. The canals were dried using paper points size 30 (Dentsply, Maillerfer, United States), and the small cotton pellet (Roeko, Whaledent GmbH, Germany) was used to clean and dry the pulp chamber [10].

#### Bioceramics dressing material preparation

The composition and the manufacturer details of the three different pulp dressing materials used in the current study has been shown in Table 2. Materials were weighed and mixed based on the instructions of the manufacturer and were applied onto the prepared samples reaching 3 mm thickness. Measurement was confirmed by a periodontal probe (#PP095-0104, Perfection Plus, South Amton, UK) by measuring the depth of the cavity before and after placement of material and digital radiograph (Romexis 2.9.2.R, Planlmeca, Helsinki, Finland). All 3 experimental groups were stored in the sterile incubator (Memmert GmbH + Co. Schwabach) under 37 ^0^ C and 100% humidity for 24 h for a complete material setting. Followed by the placement of posterior resin composite (G-aenial, Japan) without any etching and bonding, including for the samples from the positive control group.

**Table 2:** Three (3) different types of pulp dressing material involve in the study

Material	Manufacturer	Composition	Batch number
**Biodentine**	Septodont, Saint-Maur-des-Fossés Cedex, France	Powder: Tricalcium silicate, Zirconium oxide, Calcium oxide, Calcium carbonate and Colorings. An aqueous solution is composed of calcium chloride and polycarboxylate.	B28202
**MTA**	Angelus Indústria de Produtos Odontológicos S/A, Brazil	Powder: Dicalcium silicate, Tricalcium silicate, Tricalcium aluminate, Calcium tungstate Calcium oxide. Liquid: Distilled water.	60,216
**ProRoot MTA**	Dentsply Tulsa Dental, Tulsa, OK, USA	Powder: Dicalcium silicate, Tricalcium silicate, Bismuth oxide, Tricalcium aluminate and Gypsum. Liquid: Distilled water.	0000304442

**Phase 2 – Root end cutting.**

The samples from the experimental as well as positive control group were covered with a double layer of nail varnish without the occlusal surface area. Whereas group 5 (negative control), covers all the surfaces. 3 mm root length from the apical end was measured by a digital caliper before proceeding to the horizontal section using Exakt Hard Tissue Cutter (EXAKT Technologies, Inc., USA), creating an open apex to mimic the immature permanent root.

### Bacterial leakage model construction

**Fig. 1:**
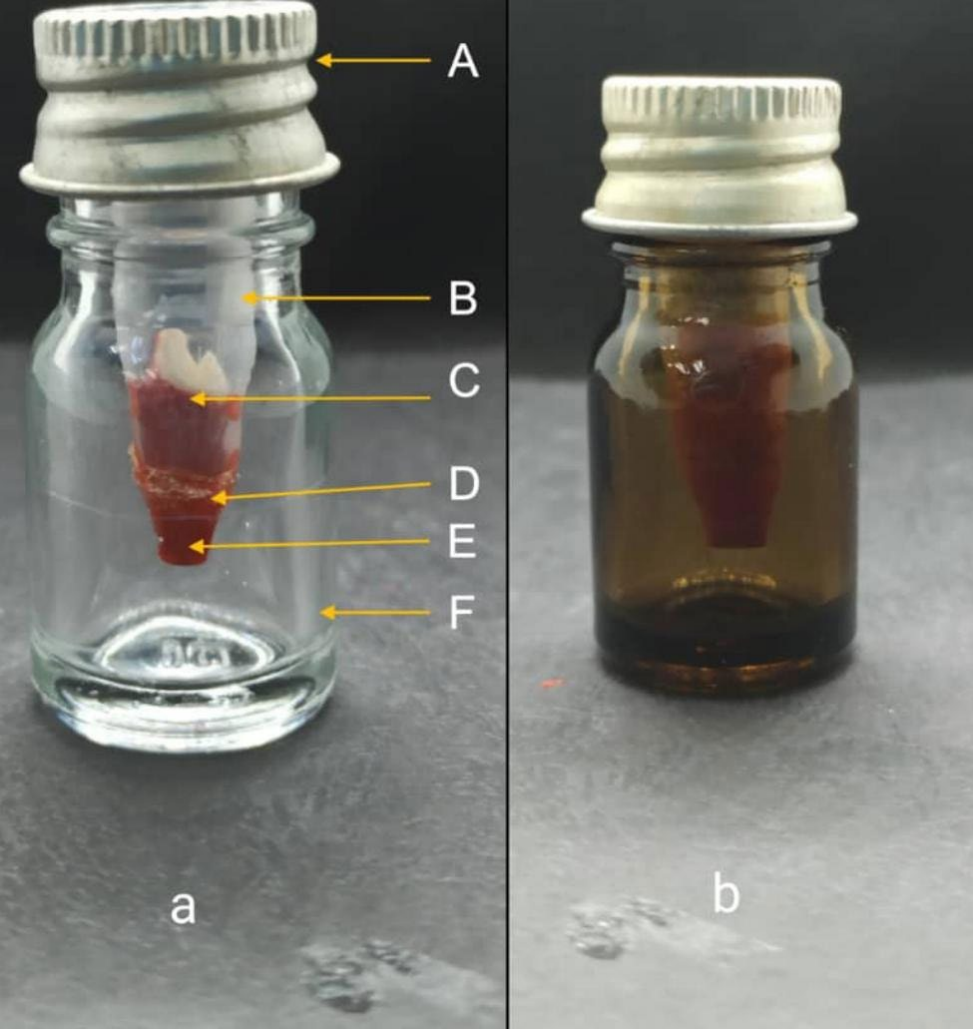
**a)** Bacterial leakage model. **(A)** Screw-cap of the lower chamber, **(B)** Upper chamber (Eppendorf tube of 1.5 ml). **(C)** Tooth inside the upper chamber, **(D)** Cyanoacrylate adhesive. (E) 2 mm protruded root. **(F)** Lower chamber (5ml clear glass bottle). **b)** Bacterial leakage model after gamma rediation

The bacterial leakage model was adapted from Torabinejad M. et al. and Lertmalapong P. et al. [8, 12] with modification using a clear screw-capped glass bottle. The upper chamber contains microcentrifuge tubes of 1.5ml (Sigma-Aldrich, St Louis, United States). One 50% of the sample was placed inside this tube, while the other 50% protruded from the bottom of the tube. Cyanoacrylate adhesive (3 M, US) was applied to seal the place between the sample and the tube wall. The lower chamber is represented with a 5 ml clear glass bottle (Fig. 1). The models were then sterilized through gamma radiation [13].

### Bacterial leakage test

The lower chambers were filled with freshly prepared BHI broth (Brain Heart Infusion) (Sigma-Aldrich, USA), with associated thickness to make sure approximately 2 mm root tip was immersed in it. [14] All the prepared samples and models were incubated in 100% humidity at 37 ^o^ C for 24 h to mimic the oral environment. *Enterococcus faecalis TCC* 23,125 was selected and the colonies from blood agar media were carefully transferred to the BHI broth by using the stick (Loop), followed by incubation in 100% humidity at 37 ^o^C for 24 h for the bacteria to grow. The growth colonies will later transfer into the McFarland tube to adjust the density of 0.5 McFarland standard under calibrated densitometer (Buch & Holm, Herlev, Denmark). The bacterial broth in an aliquot of 500µL to 1000µL was poured into the upper chamber on Day 1. The bacterial leakage models were incubated in 100% humidity at 37 ^o^C throughout the experiment. The bacterial broth of the upper chamber was replaced with the freshly prepared broth to confirm bacterial viability every 48 h during 25 days of the experiment.

Gram staining and Pyrrolidonyl arylamidase (PYR) were performed simultaneously on every sample to ensure the presence of bacteria. Bacterial survival analysis was conducted to compare the time of microleakage occurred. A Kaplan-Meier plot and a One-way ANOVA test were used to visualize the results.

### Scanning electron microscope (SEM) for marginal adaptation

A single tooth from the experimental group was randomly selected after the completion of 25 days of incubation in 100% humidity and at 37 °C. Each sample was vertically sectioned into half by Exakt Hard Tissue Cutter (EXAKT Technologies, Inc, USA). They were socked in a series of different concentrations of aqueous ethanol of 70%, 80%, 90%, 95%, and 100% for 5 min each to eliminate all remaining bacteria. [15]. The prepared samples were examined under 15.0 kV SEM 2000 magnifications to analyze the space and gap between materials and tooth structure. Microphotographs were captured and the gap measurement was recorded. One-way ANOVA as well as Post Hoc analysis were carried out for comparison.

## Results

### Bacterial leakage test

Prevented bacterial leakage for the longest period and had the most survival time. Therefore, the significant values for the longest survival time (days) related to the lowest mean leakage time (ProRoot MTA) were Mean ± (SD) = 20.867±1.529 (days) followed by (MTA Angelus) Mean ± (SD) = 17.667±1.413 (days) and (Biodentine) Mean ± (SD) = 11.6±0.804 (days) with Log Rank (df) = 10.551 (2) the *p-value* < 0.05. Table 3 indicates the establish mean bacterial leakage time for all three experimental groups. All the materials gave the estimated number of 17.911 ± 1.062 (days).

**Table 3:** The estimated mean (days) of bacterial leaking of three different bioceramics pulp dressing material

Material	Estimate (day)	Standard error	Mean (95% CI) days		Log Rank (df)	p-value
			Lower bound (Min.) days	Upper bound (Max.) days		
**Biodentine**	11.600	0.804	10.023	13.177	10.551(2)	0.005
**MTA Angelus**	17.667	1.413	14.897	20.436		
**ProRoot MTA**	20.867	1.529	17.888	23.846		
**Overall**	17.911	1.062	15.829			

* Kaplan-Meier was applied; *Significant at the level of 0.05

**Fig. 2:**
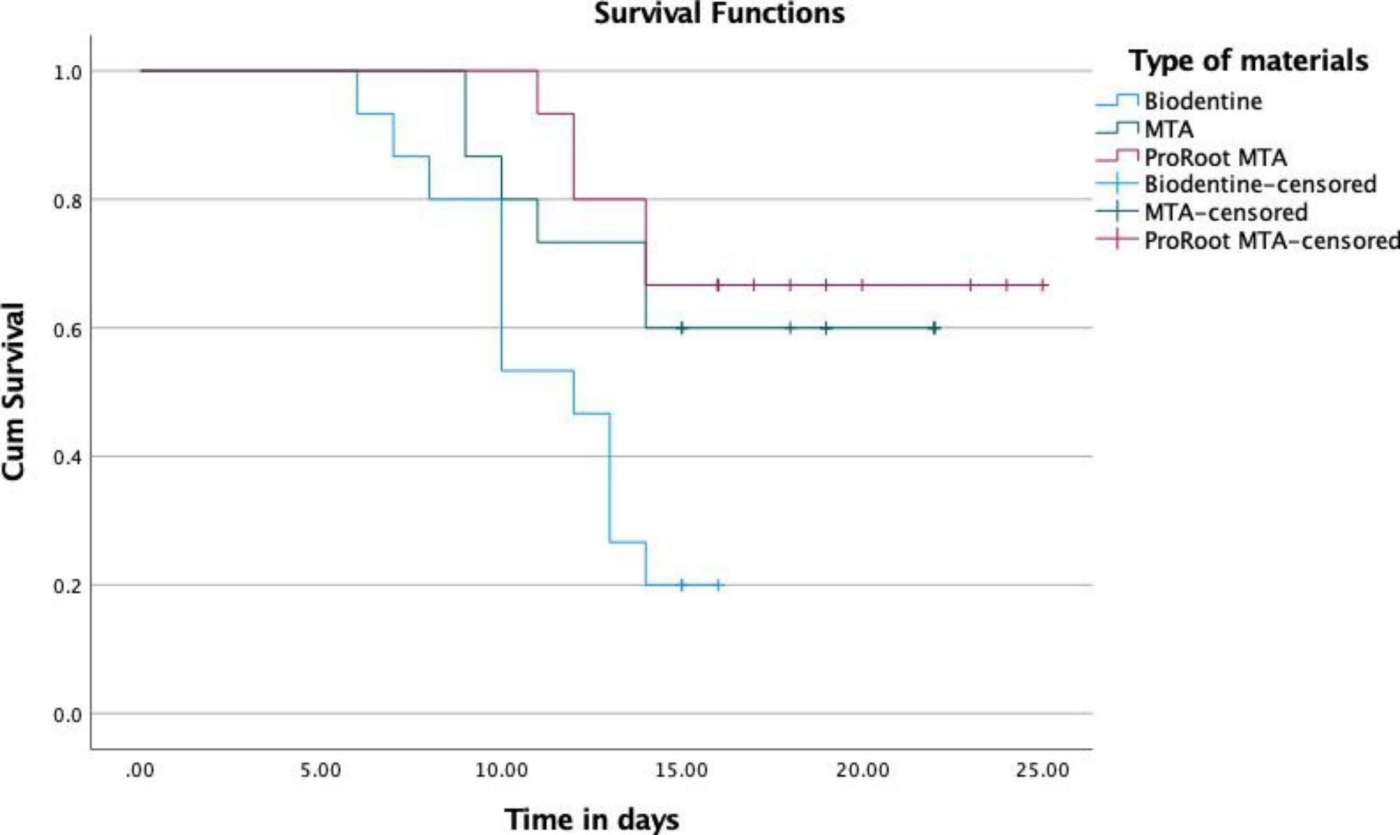
The Kaplan-Meier survival curves of three experimental groups

During 25 days of observation, the Biodentine showed a bacterial leakage from day 6 till day 16. The bacterial leakage from the MTA Angelus group started to occur on day 9 and extended to day 22. The ProRoot MTA prevented bacterial leakage for the longest period and had the most survival time, which was taken from day 11 to day 25. Figure 2 revealed all the materials showing the curve’s cutting point at the 14 days to maintain the experimental groups’ reliability. The sign of leakage from day 1 until day 14 is considered a ‘leaked group’. The samples showing any sign of leakage after day 14 are regarded as the ‘not leaked’ group. ProRoot MTA, Biodentine, and MTA Angelus-censored can be interpreted as ‘not leaked group’ at the study timeframe.

### Evaluation of marginal adaptation

The minimum significant values for the marginal adaptation (ProRoot MTA) was Mean (SD) = 1.17 (2.03) followed by (MTA Angelus) Mean (SD) = 10.9 (2.08) and (Biodentine) Mean (SD) = 16.9 (6.13) with F (2.6) = 12.295; *p-value* < 0.05.

**Fig. 3:**
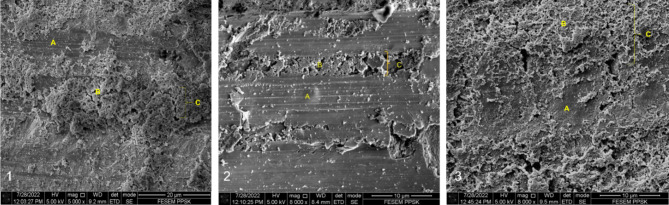
Dentinal tubule penetration of (1) Biodentine, (2) MTA Angelus and (3) ProRoot MTA on the transverse section of the tooth under SEM. (**A-** tooth surface, **B-** Material in the dentinal tubule, **C-** dentinal tubule)

The ProRoot MTA showed the least amount of gap between the dressing material and the dentinal surfaces compared to MTA Angelus and Biodentine. Table 4 shows a notable difference among the groups, which is provided as [P < 0.05; F (2,6) = 12.295]. The largest significant variance among the mean value of Biodentine vs. ProRoot MTA (Mean difference is 15.72633). And alternatively, it was discovered a slight difference between the MTA Angelus mean value vs. ProRoot MTA (The mean difference is 9.78233). That can present ProRoot MTA as a coronal pulpotomy pulp dressing medicament, which has the foremost marginal adaptation with material and dentinal walls (Fig. 3).

**Table 4:** Marginal adaptation between three different bioceramics coronal pulpotomy pulp dressing material

Bioceramics sources	Marginal adaptation Mean [23]	F (df)	Sig.
**Biodentine**	16.9 (6.13)	12.295 (2,6)	0.008
**ProRoot MTA**	1.17 (2.03)		
**MTA Angelus**	10.9 (2.08)		

*One-way ANOVA test was applied. Normality test was fulfilled. *Post Hoc analysis: Biodentine vs. ProRoot MTA – p value = 0.006. Other pairs comparison p-value > 0.05

## Discussion

To prevent bacterial leakage of coronal pulpotomy dressing material inside the oral cavity, significant properties include sealing ability and marginal adaptation. The present study evaluated the best sealing coronal pulpotomy dressing material from ProRoot MTA. The material showed the longest survival time in bacterial leakage and a very minimal marginal gap between the material and the dentinal wall. Khanna S. K. et al., (2021) used bacterial leakage test to evaluate the intraorifice sealing ability with light-cured GIC, Tetric N-Flow, along with ProRoot MTA against Proteus vulgaris and E. faecalis. However, ProRoot MTA showed better sealing ability as intraorifice coronal seal material in this study [16].

The bacterial leakage test was performed in this current study due to better biological and clinical relevance compared to the fluid filtration and dye leakage tests [8]. Previous studies conducted up to thirty to seventy-five days of the observation period for the bacterial leakage [17, 18], however our study only involved twenty-five days since there were no more bacterial leakage occurred after twenty-five days. As coronal leakage is faster than apical leakage [19].

Gamma radiation is sufficient to efficiently sterilize the upper and lower chamber samples [13]. The lower chamber changed its color from transparent to brown after sterilization. The ocular fixtures of changes in glass because of the absorption of all individual bands from the Gamma radiation [20]. In the present study, 25 kGy radiation dose was used on the samples, resulting in distortion of cyanoacrylate adhesive and plastic upper chamber in 2 samples each from MTA Angelus and ProRoot MTA, and one sample from Biodentine. An option of reducing the radiation dose might overcome the flaws and possible sample dropout. Previous studies commonly used ethylene oxide gas and autoclave to sterilize the bacterial leakage model. Ethylene oxide gas was used for the upper chamber and the autoclave procedure is used for the lower chamber [21]. In this present study, only gamma radiation is used to sterilize both chambers together in a plastic packet which reduces the chance of contamination and maintains the sterility inside the packet.

The Kaplan-Meir survival curve showed the comparison of survival leakage time between all three experimental bioceramics. Our study proved that ProRoot MTA holds the highest survival time of 23.846 days, and MTA Angelus has 20.436 days. Whereas, the Biodentine had the lowest survival time of 13.177 days. The results were contradicted by Eggmann et al. [8, 22], where authors found that early failures of pulpotomies are reported within 1st three weeks because of bacterial intervention. The contradiction could be due to the method of the study where they used GIC as a coronal seal, compared to our study which used the composite resin. The outcome of the marginal adaptation of all three materials under SEM is aligned with the result of the bacterial leakage test. ProRoot MTA showed minimal or almost no gap between the dentinal surface and dressing material. Biodentine showed the highest gap between the dentinal surface and dressing material, followed by MTA Angelus. ProRoot MTA had the most dentinal tubule penetration under 8000x magnification. Dentinal tubule penetration depends on the physiochemical property of the bioceramics, especially the particle size (Caceres et al., 2021). This can be explained as ProRoot MTA being a modified version of MTA Angelus with a smaller particle size. The size of the particles has influenced the adaptation. The smaller the particle size, the fewer particle gaps and produce more dentinal tubule penetration (Ha et al., 2015). From a clinical aspect, ProRoot MTA takes longer to set, leading to less scope to shrink (Kim et al., 2014).

Understanding that gaps between dressing material and dentinal wall may cause leakage throughout the coronal pulpotomy procedure, leading to the re-growth of microorganisms and increased risk of infection, this type of study may help clinical practice in choosing the most appropriate coronal pulpotomy dressing material. In addition, further studies should be carried out including first permanent molars which have more prevalence as a tooth that is indicated for coronal pulpotomy in paediatric dentistry. Moreover, future studies should be directed to focus on the best-performing dressing materials with increased sample size and different types of bacteria aiming to disclose possible differences between the bioceramics pulp dressing groups.

## Conclusion

Within the limitation of the study, it was shown that ProRoot MTA would be promising and favorable to be used as coronal pulpotomy dressing material. Besides, it has the better sealing ability and marginal adaptation compared to other types of dressing materials such as Biodentine and MTA Angelus. ProRoot MTA is efficient in becoming the superior sealing material for the clinical setting.

## Data Availability

The datasets used and/or analyzed during the current study are available from the corresponding author upon reasonable request.
